# Use of Concentrated Growth Factor (CGF) in Prosthetic-Guided Reconstruction on Two-Wall Bone Defect after Cystectomy: An Alternative to Traditional Regeneration

**DOI:** 10.1055/s-0043-1768973

**Published:** 2023-06-13

**Authors:** Rocco Franco, Gabriele Cervino, Giuseppe Vazzana, Fabio della Rocca, Giulia Ferrari, Marco Cicciù, Giuseppe Minervini

**Affiliations:** 1 Department of Biomedicine and Prevention, University of University of Rome “Tor Vergata,” Rome, Italy; 2 School of Dentistry Department of Biomedical and Dental Sciences and Morphofunctional Imaging, University of Messina, Messina, Italy; 3 Private Practice in Rome, Rome, Italy; 4 Saveetha Dental College and Hospitals, Saveetha Institute of Medical and Technical Sciences (SIMATS), Saveetha University, Chennai, Tamil Nadu, India; 5 Multidisciplinary Department of Medical-Surgical and Dental Specialties, University of Campania “Luigi Vanvitelli,” Naples, Italy

**Keywords:** cystetomy, bone regeneration, platelet-derived growth factor, CGF

## Abstract

This clinical case report's objective was to describe an alternative technique executed to ensure bone regeneration after removing a cystic lesion in the upper jaw. The bone defect after the cystectomy was filled with autologous fibrin-rich clots containing concentrated growth factor (CGF). A 45-year-old female patient was presumed to have a cystic lesion with massive bone destruction on the vestibular and palatal walls between teeth 2.2 and 2.3. CGF was applied to fill the gap to promote the development of the bone. The tooth was asymptomatic and repair was still increasing steadily after a year, according to the results of the clinical and radiological follow-up assessment. This article describes a different way to treat a two-wall defect involving both the palatal and buccal bone, after removing a cystic lesion, with the use of CGF as an equivalent to the traditional use of autologous or heterologous bone. A promising substance for bone repair is CGF fibrin, which may encourage the growth of new bone in jaw deformities and promote bone tissue healing.

## Introduction

The range and severity of the defect have a significant impact on the bone regeneration processes when the coagulum formation process is not hampered. Small cystic abnormalities often heal within a year on average, whereas medium-sized and big cystic defects take longer to heal, taking anywhere from 2 to 5 years. 1 After removing a cystic formation, the surgical wound is generally closed for first intention and immediately the blood clot fill the bone defect. Subsequently the clot undergoes a retraction and there is an extrusion of serum that determines the formation of a space between the clot and the wall of the bone defect. This is considered a delicate moment, since the conditions for microbial proliferation can be created in the bone space formed by the elimination of the pathological process as well as the protrusion of the vascular endothelium can be altered. For this reason, several methods have been proposed over the years to improve blood clot stabilization and to protect primary wound healing. 2 3 Because autogenous bone grafts have osteoconductive, osteoinductive, and nonimmunogenic characteristics, they are regarded as the gold standard for repairing significant bony flaws. Even though it is extremely effective, there are some drawbacks, including the scarcity of bone at some donor sites, the requirement for additional operation, and donor site morbidity, which includes pain, infection, sensory loss, and hematoma formation. Due to their unlimited availability, allografts are frequently used; however, this technique has drawbacks including uneven osteoinduction, delayed resorption, high expense, and unfavorable host immune response. Growth factors are proteins contained in platelets and blood plasma that control important techniques for healing. In bone regeneration processes, they are essential for promoting cell proliferation and new angiogenesis. 3 4 The most significant growth factors are platelet-derived growth factor, VEGF (vascular endothelial growth factor), TGF (transforming growth factor), epidermal growth factor, and insulin growth factor-1 (similar growth factor insulin 1). 5 6 The first type growth factors are PRP (platelet-rich plasma) and PRGF (plasma-rich in growth factors). Prior to application, within the surgical site, PRP and PRGF need anticoagulants and thrombin or calcium chloride to cause the formation of fibrin. On the other hand, PRF and concentrated growth factors (CGF) do not need any chemical additives for platelet activation and fibrin polymerization. CGF was developed by Sacco in the year 2006. 7 The CGF is a useful tool for bone regeneration, which may encourage the growth of new bone in jaw defects and aid bone tissue recovery. CGF has been increasingly associated with the PRF since, in comparison to it, it has shown to have higher traction resistance, more adhesive strength, and better viscosity characteristics. The use of the patient's own autologous fibrin is not typically associated with side effects, is not an expensive treatment method, and it is efficient and easy to use. 1 It has been observed that by exploiting a constant temperature and varying the centrifugal force technique with acceleration and deceleration, it is possible to activate the α-granules of the platelets and to create autologous blood products that were more concentrated in growth factors than PRF and hematopoietic stem cells (CD34+ cells). 8 9 10 A promising substance for bone repair is CGF fibrin, which may encourage the growth of new bone in jaw gaps and promote bone tissue recovery. At constant temperature, a variable-speed centrifugation method with physical accelerating and decelerating was employed. Compared with PRF and hematopoietic stem cells (CD34+ cells), autologous blood products produced by completely stimulating α-granules in platelets have higher amounts of growth factors. 3 11 CGF, by stimulating the degranulation of the α-granules of the platelets, plays a fundamental role in the initial stages of wound healing. 12 Furthermore, in comparison to other platelet-based preparations, it has been discovered that CGF contains more growth factors, and unlike PRP, CGF does not dissolve quickly after application. 13 The CGF could release growth factors for at least 13 days, according to Feigin and Shope. 14

### Objective

The aim of this article is to describe a different way to treat a two-wall defect involving both the palatal and buccal bone, after removing a cystic lesion, with the use of CGF as an alternative to traditional use of autologous or heterologous bone. The aim of this article, therefore, is also to evaluate how platelet preparations can be a useful aid for bone regeneration as demonstrated in this technical note.

## Technical Note

**Fig. 1:**
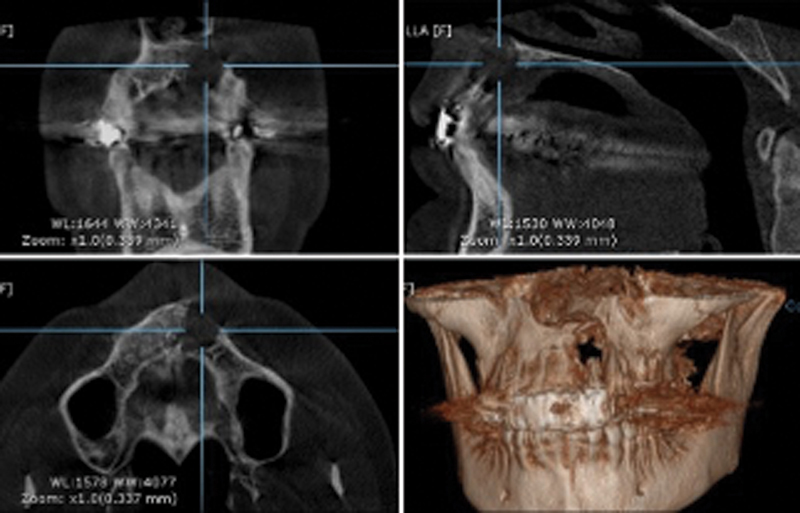
Preoperative computed tomography scan.

**Fig. 2:**
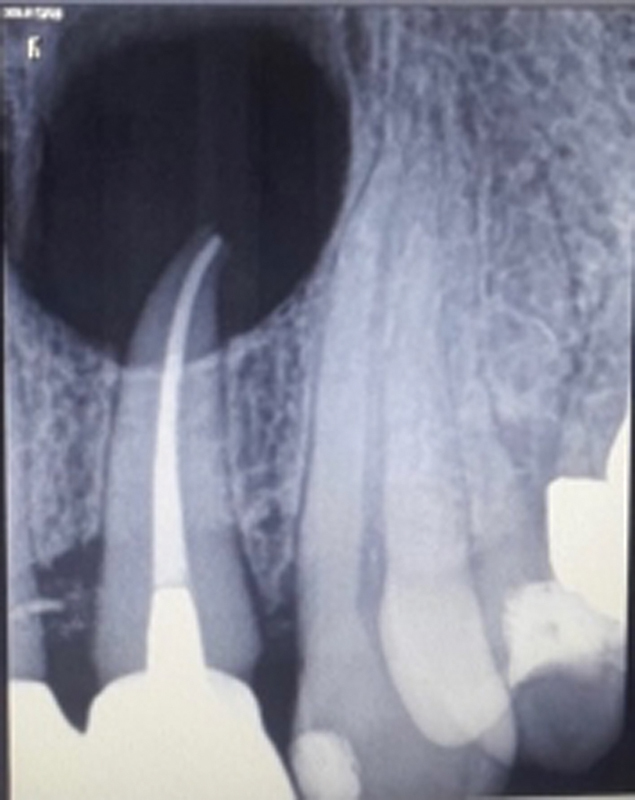
Periapical rx on #22 taken after root canal therapy.

A 45-year-old female was presented with discomfort and enlargement toward the front of the upper jaw. Clinical discoveries revealed uncomfortable and fluctuant edema in the lower vestibule. The color and hydration of the mucosa above the swelling area were both normal. Orthopantomography and cone beam computed tomography scans were performed after the examination, and they confirmed the presence of a distinct oval radiolucency in the anterior part of the upper jaw ( Fig. 1 ) that indicated extensive bone destruction on the vestibular and palatal walls between teeth 2.2 and 2.3. Before surgery, the patient received complete information about the protocol of the treatment and individually validated the consent form. An oral antibiotic (Augmentin 1 g) was administered starting the day before the procedure and continuing for 5 days after. An antiseptic mouthwash containing 0.2% chlorhexidine digluconate was used to reduce the bacterial load, and then under local anesthesia, a vestibular triangle flap access was drawn, with one vertical and one horizontal incision, the latter of which was made distally to tooth 1.1. Endodontics on 2.2 was done 1 week earlier ( Fig. 2 ).

**Fig. 3:**
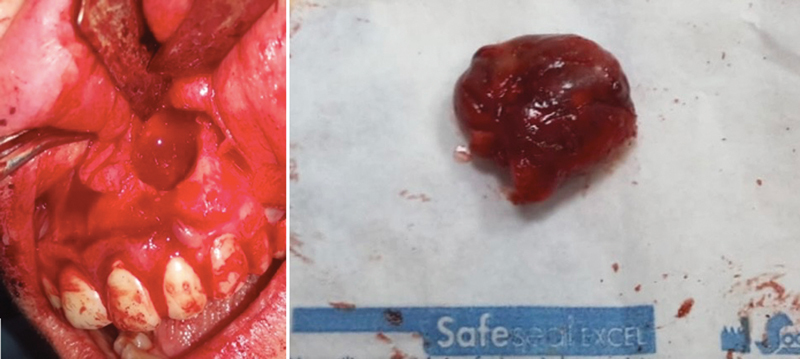
Resulting bone defect after cystectomy.

A full-thickness flap was lifted and continuously irrigated to avoid the periosteum's dehydration. It was easy to see after flap elevation that the buccal bone had been fenestrated. A mild osteotomy allowed to improve the access view and to perform the cystectomy. On the affected tooth 2.2 a root resection was done ( Fig. 3 ). Two fibrin-rich blocks with CGF were inserted to repair the ensuing bone defect, totally closing the bone cavity.

**Fig. 4:**
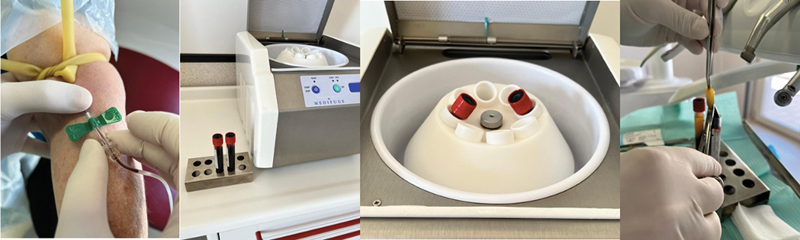
Preparation of concentrated growth factor.

**Fig. 5:**
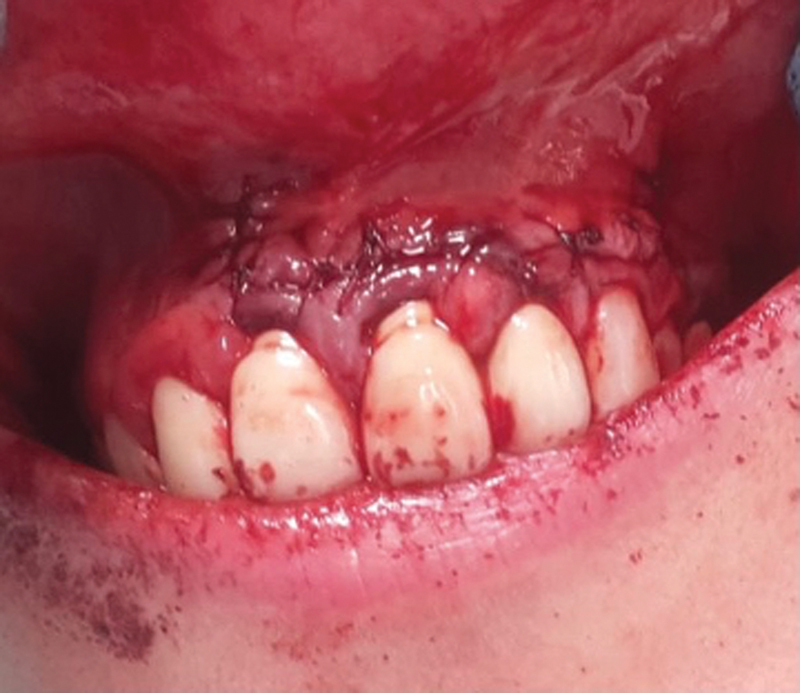
After surgery.

Patient's peripheral venous blood (20 CC) was taken from patient's vein and was used to fill two tubes (without anticoagulant), each of 10 mL. The CGF was made in accordance with the manufacturer's specifications (Silfradent, Medifuge MF200, Via G. di Vittorio 35/37, 47018 S. Sofia (FC), Italy). Four layers have been observed from the bottom to the top of each tube at the end of the procedure: the red blood cells layer, the growth factors and stem cell layer (CGF), the buffy coat layer, and the serum layer (Platelet-Poor Plasma). We removed the CGF layer with sterile surgical cutters ( Fig. 4 ). The CGF clot is then compressed to a thickness of 1 mm in a unique box. Next, the CGF is positioned above the desired area. The flap was mobilized and made easier to reposition performing periosteal incisions, which also helped to reduce muscular tension. Once again in their original location, the reflected tissues were stitched with a resorbable 5–0 suture ( Fig. 5 ; Ethicon Inc., Piscataway, New Jersey, United States). Hard, heated foods should not be eaten. For the next 10 days, it was recommended to the patient to rinse with mouthwash twice daily using 0.2% chlorhexidine digluconate. Nonsteroidal analgesics (ketoprofen) were recommended for pain relief if needed and steroidal ones (Bentelan 1 mg tablet) with decreasing dosage were prescribed after the surgical procedure, for swelling control. Seven days following surgery, the sutures were removed. Clinical examination of the tooth revealed no symptoms after a year of observation, and a radiographic evaluation revealed that the healing was progressing.

## Results

Platelets, fibrinogen, several growth factors, and CD34+ cells are all present in high numbers in CGF. Within a year, new bone tissue is produced, with minimal surgical problems and sufficient quality (density) and quantity. In addition, in our case there was very little bone formation and postoperative discomfort of the patient. As noted, the bone quality is also very good. Therefore, the technique of regeneration by growth factors can help to create and implement “bone quality and quantity.”

### Fi-Index Tool

This manuscript has been checked with the Fi-Index tool and obtained a score of (value) for the first author only on the date 20/02/2023 according to SCOPUS. 1 2 The Fi-Index tool aims to ensure the quality of the reference list and limit any autocitations. 15 16

## Discussion

Two markers (osteocalcin and bone alkaline phosphatase [BAP]) were employed to determine the growth factor fibrin concentration after CGF was introduced to the jaw's bone defect location. Osteocalcin is a protein that is produced by osteoblasts and is identified in the bone matrix. BAP is an osteoblast marker that is involved in the process of repairing bone tissue. 17 BAP and osteocalcin both represent the osteogenesis state of osteoblasts. 18 Patients with jaw fractures and bone anomalies benefited from higher levels of osteocalcin, which facilitated bone repair. CGF may be a supplementary functional healing material with osteoconductive and osteoinductive properties that is extremely biocompatible and encourages cells to secrete a few proteins that stimulate bone formation, particularly in oral surgery and implantology. 19 20 21 22 As a result, CGF would be capable of both bone guiding and osteoinduction. 23 Four weeks after surgery, histologic findings revealed that the CGF group had more preosteoblasts and osteoblasts than the control group. Additionally, the CGF group had more newly formed bone than the control group did, which may have been due to the abundance of tissue factors generated by CGF. When the CGF membrane was inserted into the gap in the jaw bone, growth factors were launched directed to undifferentiated mesenchymal cells that create a scaffold to create new bone. 24 25 26 Today, technologies and innovations thanks to major advances in digital technology enable progress in all fields of medicine. 27 28 29 30 31 32 33 34 35 36 37 Growth hormones are essential to the process of regenerating bone. Upon platelet stimulation, autologous blood-derived growth factors are released, and they are crucial for chemotaxis, mesenchymal stem cell (MSC) differentiation and proliferation, and the ability of bone cells to regenerate. Utilizing PRP has increased the popularity of growth factor clinical uses recently. PRP is well-known for being simple to use and relatively inexpensive. As a superior source of growth factors like platelet-derived growth factor, TGF-β, epidermal growth factor, VEGF, and insulin-like growth factor, PRP has been demonstrated to promote the proliferation and differentiation of MSCs. The MSCs exhibit growth factor receptors found in PRP. It has been demonstrated that using blood-derived PRP along with autologous bone marrow concentrate as a source of MSCs significantly stimulates and speeds up bone repair. Not all of the documented outcomes, however, supported the use of PRP. A small number of studies showed that PRP interfered with human osteoclast precursor differentiation and decreased the osteoinductivity of demineralized bone matrix. The effectiveness of PRP in fostering bone regeneration is thus still debatable. PRF, a variant of PRP, has properties similar to those of PRP but has better osteogenicity and a less complicated preparation procedure without the use of anticoagulants.

## Conclusion

The use of CGF as an alternative to the conventional use of autologous or heterologous bone is described in this article as a different technique to treat a two-wall defect involving both the palatal and buccal bone after the removal of a cystic lesion. CGF fibrin is a promising material for bone repair since it may encourage the growth of new bone in jaw defects and aid bone tissue recovery.
